# Exploring the sheen: a review of research advances on fruit glossiness

**DOI:** 10.3389/fpls.2025.1629039

**Published:** 2025-07-02

**Authors:** Shuchao Dong, Jiaxin Li, Jingwen Zhang, Liuxia Song, Yinlei Wang, Liping Zhao, Jie Chen, Yariv Brotman, Tongmin Zhao

**Affiliations:** ^1^ Institute of Vegetable Crop, Jiangsu Key Laboratory for Horticultural Crops Genetic Improvement, Jiangsu Academy of Agricultural Sciences, Nanjing, China; ^2^ College of Horticulture and Landscape Architecture, Yangzhou University, Yangzhou, China; ^3^ College of Horticulture, Nanjing Agricultural University, Nanjing, China; ^4^ School of Plant Sciences and Food Security, Institute for Cereal Crops Research, Tel Aviv University, Tel Aviv, Israel

**Keywords:** fruit glossiness, consumer preference, cuticle, cutin, wax, transcription factors

## Abstract

Fruit glossiness is a visually appealing trait that positively influences consumer preferences and market value. Despite its commercial importance, the biological basis of fruit glossiness has only recently gained attention. This review provides a comprehensive overview of the current understanding of fruit glossiness, with emphasis on its physiological, biochemical, and molecular underpinnings. Fruit glossiness is primarily determined by the structure and composition of the fruit cuticle, which consists of cutin and waxes. The accumulation, transport, and organization of these components dictate surface reflectivity and gloss levels. Various instrumental approaches, including gloss meters, luster sensors, spectrophotometers, and imaging systems, have been developed to objectively quantify glossiness, complementing traditional visual assessments. Advances in molecular genetics have revealed that genes involved in cuticle biosynthesis and regulation, such as *WAX2*, *CER1*, *GPAT6*, and *SHINE* family transcription factors, play critical roles in determining surface gloss. In cucumber and tomato, genetic dissection has uncovered distinct regulatory pathways involving wax and cutin metabolism, vesicle trafficking, and transcriptional control. Emerging evidence from other fruit species such as citrus, bilberry, and grape further supports a conserved yet diverse genetic architecture underlying fruit glossiness. Collectively, this review highlights the complex interplay between structural biology, environmental cues, and gene regulation in shaping fruit surface properties, and identifies promising directions for future research and crop improvement strategies.

## 1 Introduction

Fruit glossiness refers to the shiny, reflective surface appearance of fruits, which is determined by light reflection from the fruits’ outer layer (Hao et al., 2024; Xing et al., 2024). It is a key quality trait for fruit crops including grape (*Vitis vinifera*), orange (*Citrus sinensis*), apple (*Malus* × *domestica*), blueberry (*Vaccinium corymbosum*), cherry (*Prunus avium*), banana (*Musa sap.*), and pomegranate (*Punica granatum*), as well as vegetable crops in which the botanically defined fruits serve as the primary consumable part, such as tomato (*Solanum lycopersicum*), cucumber (*Cucumis sativus*), peppers (*Capsicum annuum*), and eggplant (*Solanum melongena*) (Chu et al., 2018a; Czieczor et al., 2018; Ringer et al., 2018; Wang et al., 2024). Fruit glossiness plays vital roles in shaping fruit quality and consumer preference by influencing perceptions of freshness, ripeness, texture, quality, psychological factors, and overall desirability (Huang et al., 2022; Yan and Castellarin, 2022; Yang et al., 2022; Dong et al., 2024). Glossy fruits are often perceived as recently harvested and well-maintained, indicating superior quality and taste (Zhai et al., 2022; Blanke, 2024). Previous research has shown that cucumbers exhibiting higher fruit glossiness are more attractive to consumers and tend to possess greater market value (Zhai et al., 2022; Hao et al., 2024). Likewise, the glossy covering strongly increased the appearance quality and the marketability of grape berries (Zhang et al., 2021).

From the plant’s perspective, fruit glossiness is an important trait linked to water retention, UV protection, and defense against pathogens and herbivores (Mizrach et al., 2009; Lewandowska et al., 2020; Yan and Castellarin, 2022; Hao et al., 2024). Furthermore, studies have highlighted the influence of fruit glossiness on seed dispersal mechanisms, with glossy fruits often exhibiting enhanced visibility and attractiveness to frugivores (Cazetta et al., 2009; Ordano et al., 2017).

The level of glossiness varies significantly among different fruit species and even within varieties. Some fruits, such as apple, cherry, plum, and grape, exhibit natural glossiness due to waxy cuticle or smooth surfaces (Mizrach et al., 2009; Mukhtar et al., 2014; Zhang et al., 2021). Fruit glossiness can also be enhanced or diminished by post-harvest treatments. For instance, during postharvest handling, the natural wax on the surface of citrus fruits is typically removed during washing on the packing line and subsequently replaced with a coating that often includes fungicides and additional waxes. This treatment not only prolongs shelf life but also enhances fruit glossiness (Njombolwana et al., 2013; Babarabie et al., 2024). For growers and retailers, enhancing the glossiness of fruit products through pre-harvest or post-harvest treatments, such as bagging of low-density polyethylene or coating of epigallocatechin-3-gallate (EGCG), lemongrass oil, and carnauba wax (E 903), can improve the product’s visual appeal and extend its shelf life (Kim et al., 2014; Singh et al., 2016; Ali et al., 2021; Sapna et al., 2024; Wu et al., 2024).

Fruit glossiness is influenced by various factors, including genotypes, environmental conditions during fruit development, and post-harvest treatments (Mahjoub et al., 2009; Kim et al., 2014; Liu et al., 2015; Yang et al., 2021; Prasad et al., 2024). Recent research has illuminated the intricate mechanisms underlying fruit glossiness. Studies employing advanced imaging techniques, such as confocal microscopy and scanning electron microscopy, have elucidated the role of epicuticular wax crystals and cuticular folds in shaping the glossy appearance (Liang et al., 2021; Zhang et al., 2021; Gao et al., 2023; Liu et al., 2023). Moreover, molecular studies have identified key genes and enzymes involved in wax/cutin biosynthesis and deposition, shedding light on the genetic basis of fruit glossiness in cucumber, tomato, and citrus (Liang et al., 2021; Yang et al., 2021; Zhai et al., 2022; Liu et al., 2023). Agricultural research has explored strategies to modulate fruit glossiness through genetic manipulation or agronomic practices, aiming to improve marketability and shelf life (Kim et al., 2014; Singh et al., 2016). Additionally, consumer studies have delved into the psychological factors influencing fruit preference, with glossiness emerging as a key determinant of perceived freshness and quality (Blanke, 2024; Dong et al., 2024).

Understanding the regulatory mechanisms of fruit glossiness has broad implications for growers, breeders, and the food industry. In this review, we synthesize existing research on the factors influencing fruit glossiness, methodologies for gloss measurement, and the underlying gene regulatory mechanisms. Additionally, we discuss related aspects to provide a comprehensive reference for future studies on fruit glossiness and its potential applications in the molecular breeding of fruit and vegetable crops.

## 2 Fruit glossiness is mainly and directly determined by cutin and wax of cuticle layer

Fruit glossiness is primarily governed by the composition and structure of the fruit’s cuticle layer, mainly cutin and wax (Shi et al., 2013; Hao et al., 2024). The composition of cutin and cuticular waxes, including their quantity and distribution, largely determines the degree of glossiness on the fruit’s surface (Kimbara et al., 2012; Liang et al., 2021). Most of the glossy or matt fruit phenotypes have been reported to be related to the structure and production of wax and cutin (Shi et al., 2013; Liang et al., 2021).

**Figure 1:**
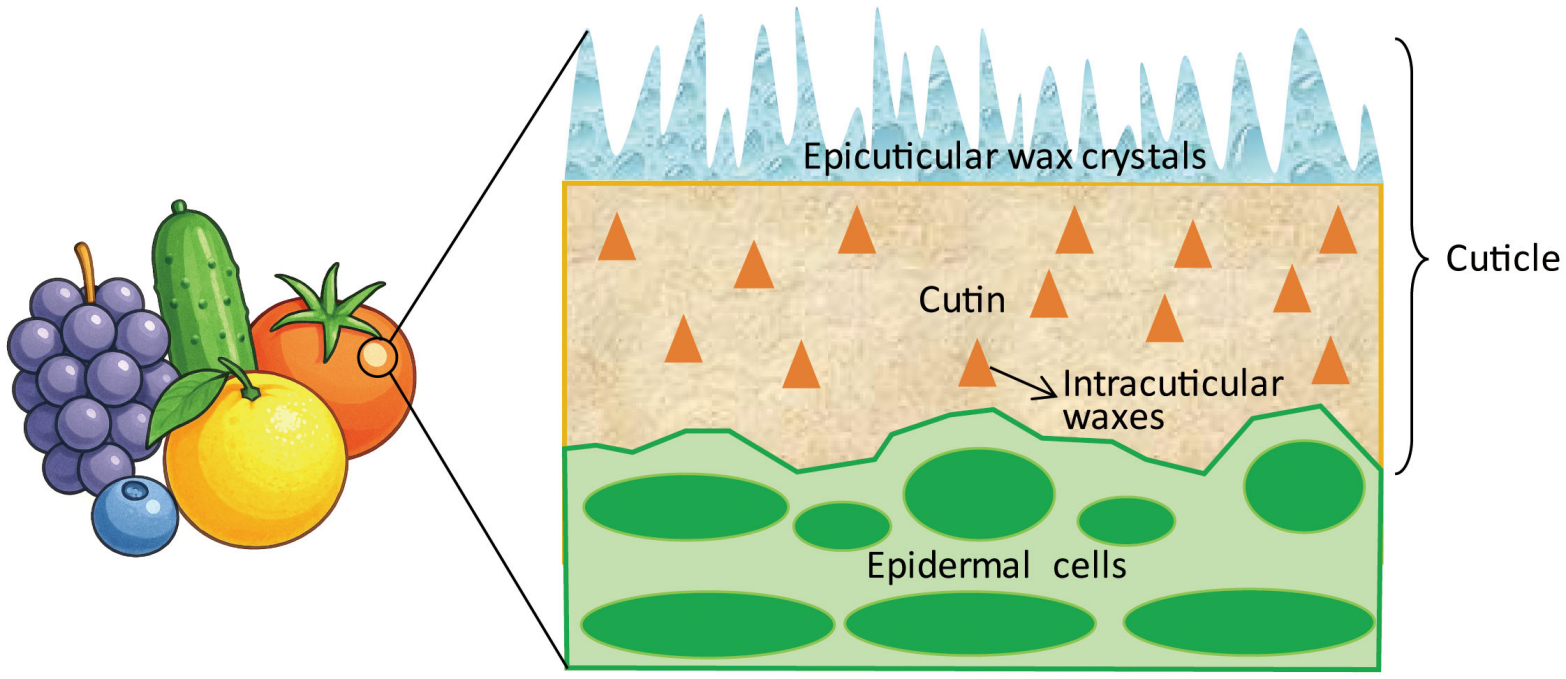
A simplified scheme of the fruit cuticle structure. The cuticular layer is a distinct zone on the epidermis layer, consisting of epicuticular wax crystals, and cutin embedded with intracuticular waxes.

Variations in wax structure, from smooth to crystalline, affect light reflection, which in turn determines the level of glossiness (Zeisler-Diehl et al., 2018). As shown in **Figure 1**, cuticular waxes present on the fruit surface are generally classified into two major types: epicuticular and intracuticular (Jetter and Kunst, 2008; Koch and Ensikat, 2008; Hao et al., 2024). Epicuticular waxes typically form two- or three-dimensional structures ranging from hundreds of nanometers to several micrometers in size. These microstructures influence light reflection at the cuticle surface, thereby playing a critical role in determining fruit glossiness (Koch and Ensikat, 2008). Intracuticular waxes are embedded within the mechanically resistant layer of cutin polymer matrix (Buschhaus and Jetter, 2011). Plant cuticular waxes are primarily composed of very-long-chain fatty acids (VLCFAs, typically C_20_–C_34_) and their derivatives, including alkanes, aldehydes, primary and secondary alcohols, ketones, and esters. In addition, they may contain secondary metabolites such as triterpenoids, sterols, tocopherols, and phenolic compounds (Bourdenx et al., 2011; Yeats and Rose, 2013; Trivedi et al., 2019; Hao et al., 2024). The composition of cuticular waxes vary distinctly among cultivars and fruit species (Yeats et al., 2012; Trivedi et al., 2019). Analysis of matt and glossy mutants showed that different waxes were differentially accumulated in those mutants (Liang et al., 2021; Zhai et al., 2022; Hao et al., 2024).

Cuticular waxes are biosynthesized in the epidermal cells of fruit peel (Trivedi et al., 2019). Firstly, aliphatic wax constituents were produced *de novo* with C_16_ and C_18_ fatty acids in plastids, which are subsequently elongated to C_20_–C_34_ VLCFAs in the endoplasmic reticulum (ER) by the fatty acid elongase (FAE) complex. Within FAE complex, β-ketoacyl-CoA synthase (KCS) serves as the rate-limiting enzyme (Leide et al., 2007; Kunst and Samuels, 2009; Yeats and Rose, 2013). The resulting VLCFAs are further processed through two main pathways: the acyl reduction pathway, which yields esters and primary alcohols, and the decarbonylation pathway, which generates alkanes, aldehydes, ketones, and secondary alcohols (Kunst and Samuels, 2009). In parallel, wax triterpenoids and sterols are synthesized from squalene, a product of the mevalonate pathway, which undergoes further modifications to yield diverse compounds (Thimmappa et al., 2014; Trivedi et al., 2019). Alterations in wax biosynthetic pathways, whether caused by mutations in genes encoding wax biosynthesis enzymes or by changes in their expression levels, can result in variations in fruit glossiness.

In addition to wax, cutin is another major component of the cuticle layer on the fruit surface. It is an insoluble polyester primarily composed of C_16_/C_18_ hydroxy fatty acids. Fruit glossiness has been shown to be closely associated with the content and composition of cutin (Shi et al., 2013; Bres et al., 2022). For example, the glossy genotype PI 257145 exhibited a 6-fold higher cutin content compared to the matt genotype PI 224448 in hot peppers (*Capsicum chinense* Jacq.). Metabolomic analysis revealed that 12 distinct cutin monomers accumulated at higher levels in PI 257145. Among them, 10,16-dihydroxy hexadecanoic acid was present at more than 9-fold the concentration observed in PI 224448 (Natarajan et al., 2020). The biosynthesis, transport, and polymerization of cutin monomers have been well characterized (Chen et al., 2025). Briefly, C_16_/C_18_ fatty acids (C_16_/C_18_ FA) originating from plastids are converted into their corresponding coenzyme A derivatives (C_16_/C_18_-CoA) by LACS (long-chain acyl-coenzyme A synthase) isozymes in the endoplasmic reticulum. These are then hydroxylated by CYP86A (ω-hydroxylation) and CYP77A (midchain hydroxylation) to form di-/tri-hydroxylated derivatives (Petit et al., 2017). Glycerol-3-phosphate acyltransferase (GPAT) converts these into 2-monoacylglycerols such as 2-MHG (Yeats et al., 2012), which are transported by ABC transporters (Elejalde-Palmett et al., 2021; Chen et al., 2025) and polymerized into cutin by cutin synthases, such as SlCUS1/CD1 and SlGDSL1 (Girard et al., 2012; Yeats et al., 2014).

It is worth noting that a waxy surface is often associated with reduced glossiness in many species, making waxiness and glossiness inversely related traits (Liang et al., 2021; Zhang et al., 2021; Hao et al., 2024). For example, fruit glossiness in cucumber is largely influenced by the presence of bloom, also known as wax powder, which appears as a gray-white frosty layer on the fruit surface and significantly reduces glossiness (Zhai et al., 2022; Hao et al., 2024). Previous studies have demonstrated that repression of wax production leads to a glossy fruit phenotype (Zhai et al., 2022). The cucumber line *3413* exhibited reduced wax accumulation and enhanced glossiness (Wang et al., 2015b). Similarly, a study in grape showed a negative correlation between fruit glossiness and surface wax density across different cultivars (Zhang et al., 2021). In tomato, reduced accumulation of cutin and wax in the fruit cuticle has also been associated with a glossier surface phenotype (Shi et al., 2013), further supporting the conserved role of wax load on fruit surface in determining fruit glossiness.

## 3 External and internal factors affecting fruit glossiness

**Figure 2:**
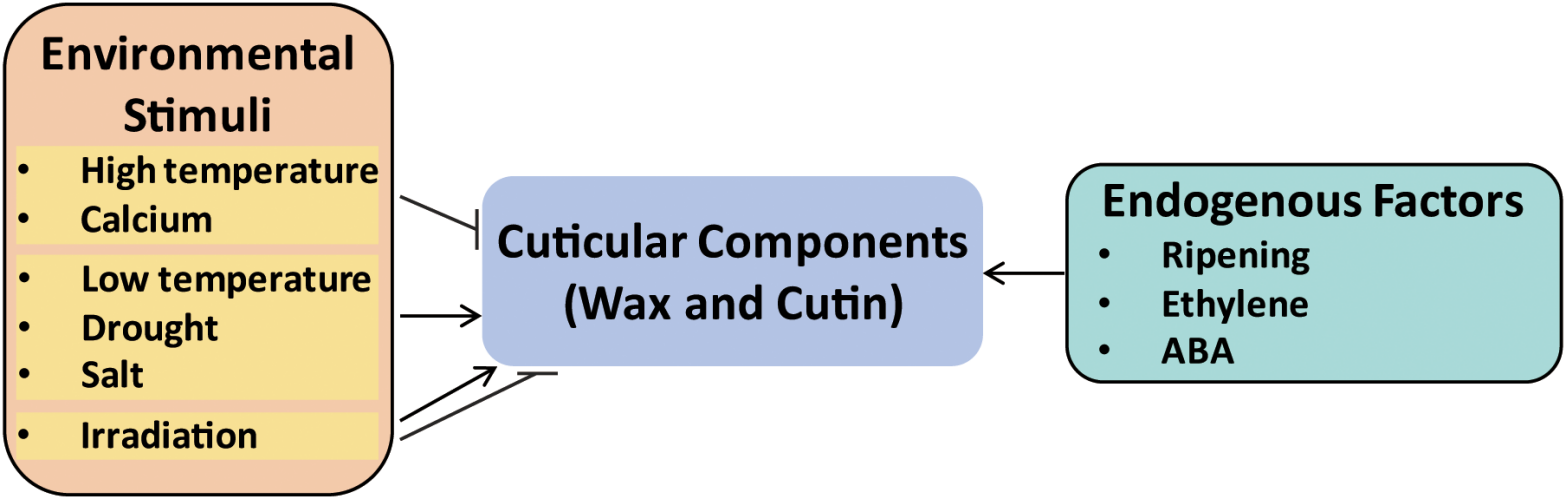
External and internal factors affecting fruit glossiness. Environmental factors such as temperature, drought, salt, UV-B irradiation, and calcium treatments influence cutin and wax biosynthesis. Hormones like ethylene and ABA also regulate cuticle formation.

Fruit glossiness is primarily determined by the deposition of cuticular components, particularly wax and cutin, on the fruit surface (**Figure 1**). Variations in the accumulation of these components can lead to noticeable changes in fruit glossiness (Mukhtar et al., 2014; Liu et al., 2023; Hao et al., 2024). The regulation of wax and cutin biosynthesis by external stimulus has been reported in various crop species, including apple, tomato, grape berry, rice, and cucumber (Charles et al., 2008; Xue et al., 2017; Trivedi et al., 2019). As shown in **Figure 2**, environmental stimuli and endogenous factors exert distinct influences on cuticle development. Temperature has been shown to affect wax production, with many plants exhibiting increased wax accumulation under lower temperature conditions. For example, low temperature was shown to increase the thickness of the cuticular wax layer and the content of alkanes in *Malus* crabapple, thereby reducing fruit glossiness (Hao et al., 2017). However, in contrast to these findings, wax content in both blueberry and “Red Fuji” apple has been shown to decrease significantly during postharvest cold storage (Lara et al., 2014; Chu et al., 2018b). Whereas, an earlier study demonstrated that heat treatment at 38°C for four days was found to alter the cuticle structure and reduce wax accumulation on the surface of ‘Golden Delicious’ apples (Lurie et al., 1996).

Drought and salt have been reported to induce cuticular wax biosynthesis in cucumber fruit, typically resulting in reduced glossiness (Wang et al., 2015a). Exogenous calcium treatment has been shown to enhance the glossy appearance of grape berries by reducing cuticular wax accumulation (Martins et al., 2021). Although the impact of irradiation on fruit glossiness remains underexplored, a previous study revealed that exposure to moderate to high doses of UV-B irradiation promoted the formation of glossy layers on the adaxial surfaces of cotyledons, while also affecting both the quantity and composition of surface wax (Fukuda et al., 2008). Additionally, exposure to irradiation has been associated with a thicker cuticular wax layer in several plant species (Shepherd and Wynne-Griffiths, 2006; Trivedi et al., 2019). Taken together, stress conditions generally enhance the production of cutin and wax, which in most cases leads to a reduction in fruit glossiness.

In addition to environmental cues, the biosynthesis of wax and cutin is also regulated by multiple endogenous factors (Trivedi et al., 2019; Lewandowska et al., 2020). For example, total wax content increased significantly during fruit ripening in blueberry cultivars such as “Brightwell” (*Vaccinium ashei*) and “Legacy” (*Vaccinium corymbosum*) (Chu et al., 2018b). In tomato, ethylene has been shown to play a central role in promoting cutin deposition, thereby influencing fruit glossiness (Bres et al., 2022). Abscisic acid (ABA) has also been implicated in this process, as ABA induced cuticular wax biosynthesis in cucumber fruit (Wang et al., 2015a), and regulates both cutin and wax biosynthesis in the tomato fruit epidermis, ultimately affecting surface gloss (Liang et al., 2021).

## 4 Methods of measuring fruit glossiness

**Figure 3:**
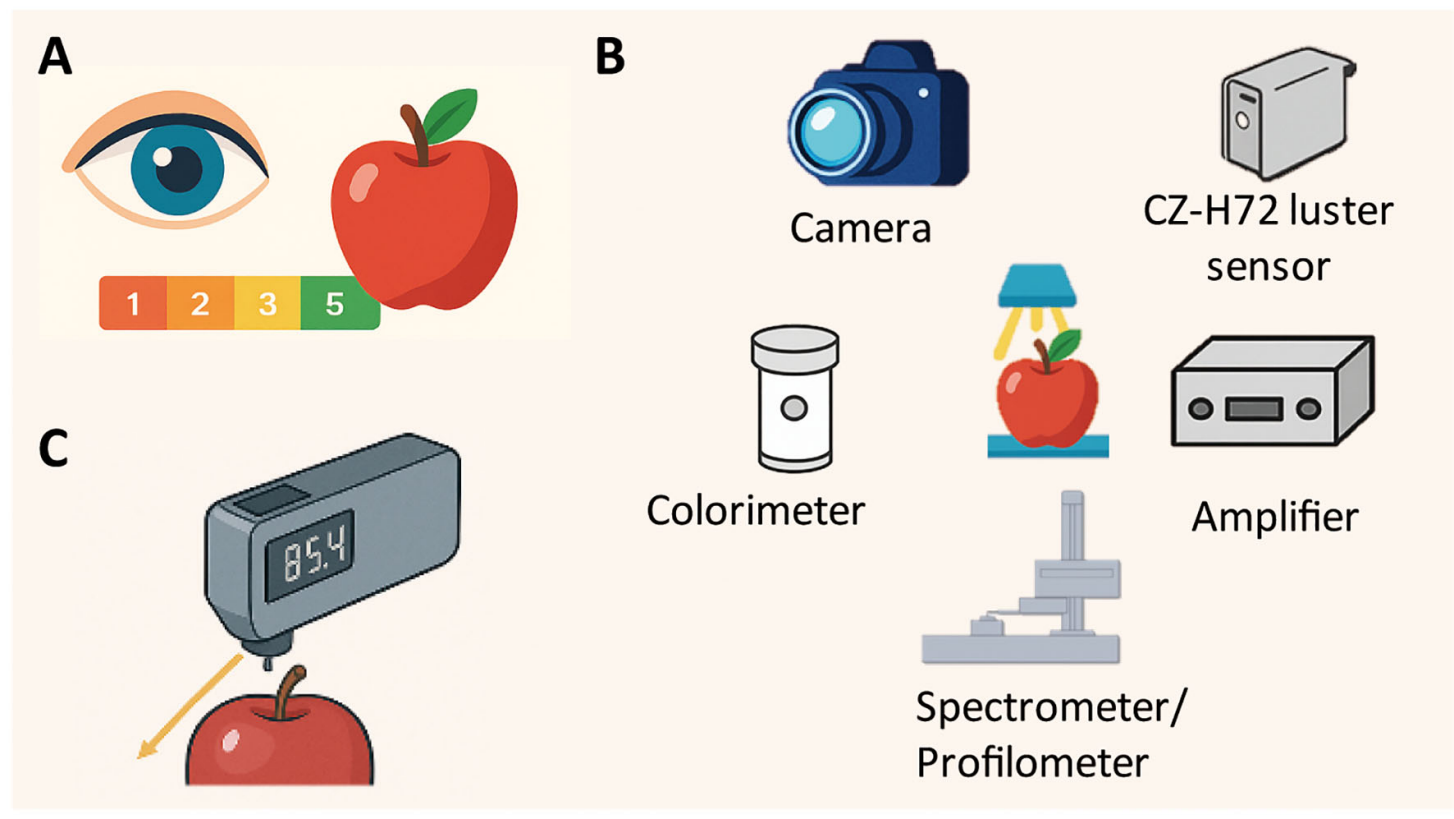
Three main approaches for evaluating fruit glossiness. **(A)** Visual inspection, where glossiness is assessed subjectively by eye, often using a qualitative scale (e.g., 1–5); **(B)** Image analysis system, incorporating instruments such as a CZ-H72 luster sensor, camera, colorimeter, amplifier, profilometer, and spectrometer to capture and quantify surface characteristics objectively; **(C)** Gloss meter, which directly measures surface gloss by detecting reflected light at standardized angles (commonly 60°) to provide precise gloss values.

Objectively, accurately, and efficiently measuring fruit glossiness has been a longstanding practical challenge. Various instruments, such as image analysis systems, spectrophotometers, and gloss meters, have been developed over the past decades to evaluate glossiness using defined measurement values under standardized conditions (**Figure 3**) (Mizrach et al., 2009; Mendoza et al., 2010; Dong et al., 2024). These techniques offer distinct advantages depending on the specific research objectives and the physical characteristics of the fruit.

In some recent studies, fruit glossiness was evaluated through visual inspection. The glossiness was qualitatively scored as either glossy or matt (Petit et al., 2014; Zhai et al., 2022). Alternatively, a 1–5 scale was used for visual assessment of fruit glossiness (Prohaska et al., 2024). While this method was able to rapidly differentiate between glossy and matt fruits, it was inherently subjective, as assessments depended on individual perception and environmental lighting conditions. Moreover, visual evaluation lacks the precision required for quantitative analyses, making it unsuitable for large-scale studies such as genome-wide association studies, which demand accurate and reproducible measurements.

A previous study developed a gloss imaging system comprising a light source, camera, condenser, and filter to quantitatively analyze the surface gloss of fruits and vegetables (Mendoza et al., 2010). Althaus and Blanke (2020) designed a specific instrument to measure surface glossiness of pepper fruits using a CZ-H72 luster sensor (Keyence, Co., Osaka, Japan) coupled with a colorimeter, a spectrometer and a profilometer type VR-5200 (Keyence) to obtain RGB images (Althaus and Blanke, 2020). A similar research was also conducted using CZ-H72 luster sensor and profilometer to measure the fruit glossiness in banana (Ringer et al., 2018). Mukhtar et al. (2014) assed fruit glossiness of approximately 360 European plums (*Prunus domestica* L.) using a system composed of an amplifier CZ-V21AP (Keyence, Japan) and a luster sensor CZ-H72 (Keyence, Japan) (Mukhtar et al., 2014). Subsequently, Czieczor et al. (2018) applied the same method to measure the surface gloss of pomegranate fruit (Czieczor et al., 2018). The CZ-H72 luster sensor is well-suited for field applications, as it weighs only 50 g and can be conveniently powered by a smartphone or a small power bank.

The most convenient way to quantify the glossy phenotype is using a gloss meter, which was specifically designed to measure glossiness by quantifying the amount of light reflected off a surface at a specified angle. In general, larger reflection angles are typically employed to distinguish between lower-gloss objects, while smaller angles are used for high-gloss objects (Li et al., 2009). An angle of 20° is most effective for high-gloss materials, whereas 75° or 85° is optimal for low-gloss materials. The 60° geometry provides the best overall correlation with visual assessments and has been widely adopted by the American Society for Testing and Materials (ASTM) in ASTM method D523 for a variety of materials (Mizrach et al., 2009).

Different types of gloss meters have been used in numerous studies. For instance, Ward and Nussinovitch (1996) compared a curved surface glossmeter and a flat surface glossmeter (Triple Angle Novo-Gloss, Rhopoint Instrumentation Ltd, Germany) for measuring the glossiness of fruit peels in eggplant, apple, and apple. Interestingly, the two types of gloss meter produced differing results when assessing the glossiness of tomato fruits before and after wax removal. A recent study showed that the glossiness values of tomato fruits were measured by a micro-hole gloss meter NHG60M (Shenzhen 3nh Technology Co., Ltd, Shenzhen, China) at 60° angle (Dong et al., 2024). Mizrach et al. (2009) used another gloss meter (Elcometer 400 Novo-Curve, Nova, MI, USA) to evaluate the gloss appearance of apples at a 60° angle (Mizrach et al., 2009). Gloss levels of cucumber fruits were measured using either an XA6 Curve Gloss Meter (JND, Shanghai, China) or a HYD-09 glossmeter (Yang et al., 2022; Zhai et al., 2022). In contrast to the 60° angle measurements, a previous study used a colorimeter (HP-200) to measure the fruit glossiness of cucumber, with the glossiness value represented by the *L* value, ranging from 0 (black) to 100 (white) (Gao et al., 2023). Similarly, Ren et al. (2023) analyzed fruit glossiness of cucumber using a colorimeter (CR-410) (Ren et al., 2023). It is important to note that most commercial gloss meters are designed for measuring products with flat surfaces and, therefore, are not ideal for fruits with uneven or curved surfaces. Even with a micro-hole design, high variations can occur due to the curvature of the fruit’s surface.

## 5 The molecular and gene regulatory mechanism of fruit glossiness

The molecular and genetic regulation of fruit glossiness involves complex pathways that coordinate cuticle development, the biosynthesis and transport of wax and cutin, and the modulation of surface characteristics (Liang et al., 2021; Hao et al., 2024). As presented in **Table 1**, key regulatory components include genes encoding enzymes involved in wax and cutin biosynthesis, as well as transcription factors (TFs) that orchestrate their expressions (Shi et al., 2013; Gao et al., 2023). In this review, we primarily focus on regulatory genes that have been functionally characterized. A concise summary of these mechanisms is presented below, supported by detailed citations for each gene.

**Table 1:** Regulatory genes of fruit glossiness in cucumber and tomato.

Species	Genes	Effects on fruit glossiness	Functions	Reference
Cucumber (*Cucumis sativus*)	*CsCER6*,*CsCER7*	Negative	Enhance cuticular wax accumulation.	Liu et al., 2023
	*CsWAX2*	Negative	Wax biosynthesis gene.	Wang et al., 2015a
	*CsCER1*	Negative	Enhance wax crystal accumulation.	Wang et al., 2015b
	*CsDULL*	Negative	Enhance the production and deposition of cutin and wax.	Zhai et al., 2022
	*CsZFP6*	Negative	Enhance cuticular wax accumulation.	Yang et al., 2022
	*CsSEC23*	Negative	Affect cuticle formation.	Gao et al., 2023
	*CsWIN1*	Positive	Associate with wax biosynthesis.	Zhang et al., 2019
Tomato (*Solanum lycopersicum*)	*SlHN3*	Negative	Enhance cutin and wax accumulation.	Shi et al., 2013
	*SlSHN2*	Negative	Enhance cutin accumulation.	Bres et al., 2022
	*CD2*	Negative	Enhance cutin accumulation, affect wax and cutin compositions.	Nadakuduti et al., 2012
	*SlGDSL2*	Negative	Cutin assembly and polymerization.	Petit et al., 2014
	*SlGPAT6*	Negative	Cutin biosynthesis gene.	Petit et al., 2016
	*SlPP2C*	Positive	Affect cuticle structure.	Liang et al., 2021
	*SlMIR164a*	Positive	Enhance cutin and wax accumulation.	Gupta et al., 2021

### 5.1 The regulatory mechanism of fruit glossiness in cucumber

**Figure 4:**
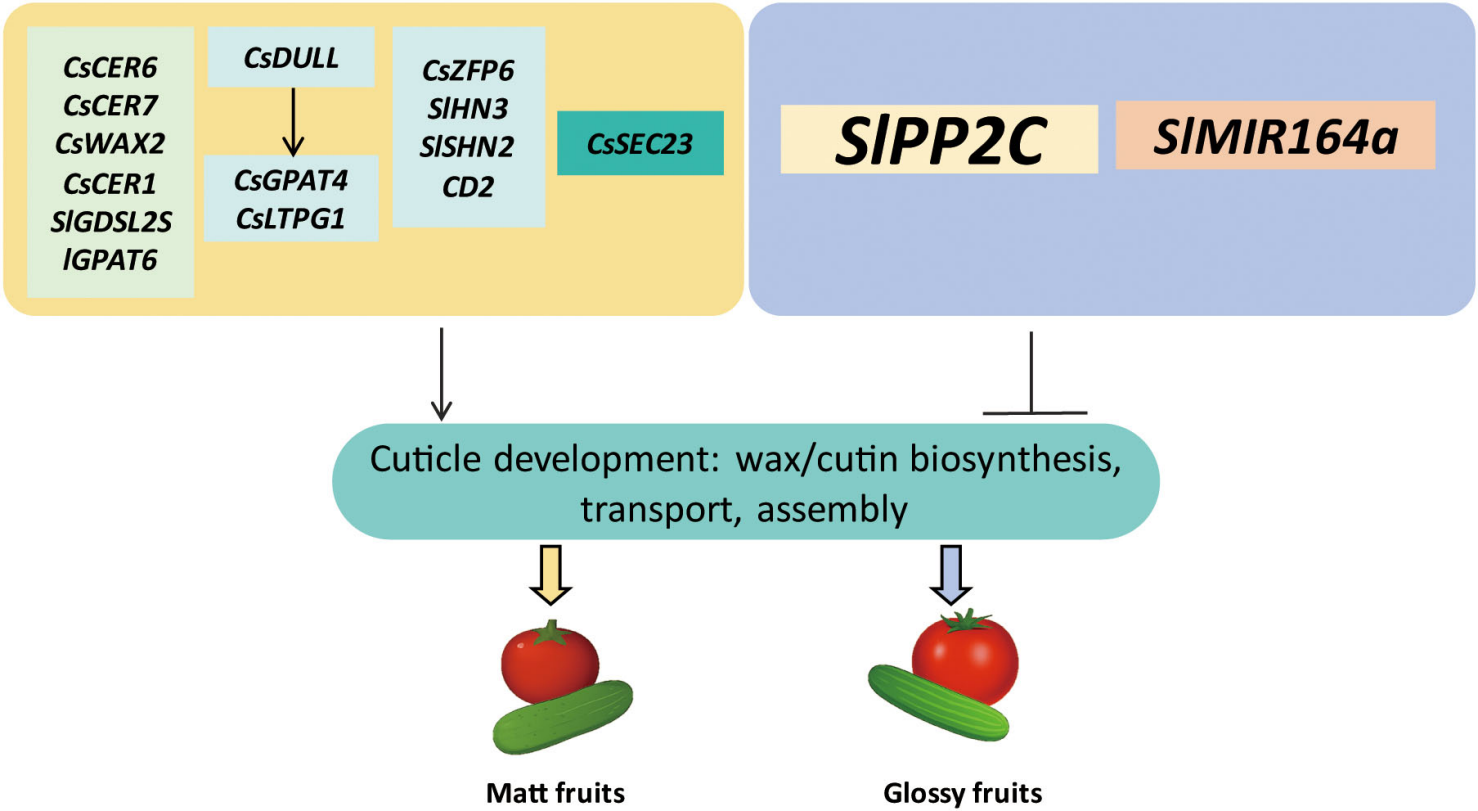
A simplified model illustrating the gene regulatory mechanisms underlying fruit glossiness in tomato and cucumber. Fruit glossiness is determined by the coordinated regulation of cuticle development, including the biosynthesis, transport, and deposition of cutin and wax components. Wax biosynthesis genes such as *CsWAX2*, *CsCER1*, and *CsCER6*, along with cutin biosynthesis genes like *SlGPAT6* and *SlGDSL2*, directly influence the production of wax and cutin. Key TFs such as *SHN*, *HD-ZIP IV*, AP2/ERF, and zinc finger proteins (e.g., *CsDULL* and *CsZFP6*) regulate the expression of genes involved in cutin and wax biosynthesis. Transport-related proteins (CsLTPG1 and CsSEC23) facilitate the movement of cuticular components to the epidermal surface. Additionally, hormonal regulators like PP2Cs (in ABA signaling) and microRNAs (e.g., SlMIR164a) modulate the expression of cuticle-related genes. These factors collectively shape the composition and organization of the cuticle, ultimately influencing the glossy or matt appearance of fruit surfaces.

The regulatory mechanism underlying fruit glossiness in cucumber has been well elucidated in recent years. Cucumber fruit glossiness is primarily determined by the quantity and composition of surface wax, including both wax and bloom (Hao et al., 2024). As shown in **Figure 4**, numerous genes involved in wax biosynthesis, transcriptional regulation, and structural organization have been identified as key regulators of fruit glossiness in cucumber (Wang et al., 2015b; Zhai et al., 2022; Gao et al., 2023).

The wax biosynthesis gene *CsCER6* and its regulatory gene *CsCER7* were shown to positively regulate cuticular wax accumulation, thereby contributing to a matt fruit phenotype (Liu et al., 2023). Similarly, CsWAX2, which catalyzes the conversion of very-long-chain (VLC) acyl-CoAs to VLC alkanes, plays a central role in wax biosynthesis (Wang et al., 2015a). The aberrant expression of *CsWAX2* in transgenic cucumbers resulted in changes to both cuticular wax deposition and the composition of cutin in the cucumber fruit. Silencing *CsWAX2* led to a glossy surface of cucumber fruits. Additionally, the expression of *CsWAX2* gene was strongly induced by cold, drought, and salinity (Wang et al., 2015a). Another wax biosynthesis gene *CsCER1*(Cucumber ECERIFERUM1) was found to be highly expressed in the matt-fruited cucumber mutant line *3401*, and expressed at low levels in the glossy mutant line *3413*. Functional analysis revealed that silencing *CsCER1* significantly reduced the formation of epicuticular wax crystals, resulting in a glossy fruit phenotype. Conversely, overexpression of *CsCER1* enhanced wax crystal accumulation, leading to a matt fruit appearance. In addition, *CsCER1* expression was shown to be inducible by abiotic stresses such as low temperature, drought, salt stress, and by the phytohormone ABA (Wang et al., 2015b).

Two C2H2-type zinc finger transcription factor have been characterized as key regulators of fruit glossiness in cucumber. A recent study identified *CsDULL*, a C2H2-type zinc finger transcription factor, as the gene associated with the *D* locus in the matt fruit mutant of cucumber. Loss-of-function mutant of *CsDULL* exhibited a glossy peel phenotype. Functional analysis revealed that *CsDULL* directly activated the expression of genes involved in cutin and wax biosynthesis and transport, *CsGPAT4* (Glycerol-3-Phosphate Sn-2-Acyltransferase 4) and *CsLTPG1* (Glycosylphosphatidylinositol-anchored Lipid Protein Transfer 1), thereby enhancing the production and deposition of cutin and wax on the fruit surface (Zhai et al., 2022). Another C2H2-type zinc finger transcription factor, CsZFP6, was shown to act as a positive regulator of cuticular wax biosynthetic genes. Loss-of-function mutants of *CsZFP6* displayed a glossier pericarp, which correlated with a significant reduction in cuticular wax accumulation (Yang et al., 2022).

Additionally, TFs of the APETALA2/ethylene-responsive factor (AP2/ERF) family have been shown to play central roles in regulating cuticle formation (Shi et al., 2013). RNA-Seq analysis by Zhang et al. (2019) identified the AP2/ERF-type transcription factor CsWIN1 as a key regulator of fruit glossiness in grafted cucumbers. Increased expression of *CsWIN1*, along with wax biosynthetic genes, was associated with the glossier appearance of the cucumber pericarp in grafted plants (Zhang et al., 2019). However, a more recent study showed that grafting cucumber onto pumpkin (*Cucurbita moschata*) rootstock significantly reduced wax accumulation and enhance fruit glossiness (Ren et al., 2023). The results of *CsWIN1* contrast with other studies in cucumber, which have observed a negative correlation between wax production and fruit glossiness. This suggests that the relationship between wax accumulation and surface glossiness might be tissue-specific or influenced by additional regulatory components.

Moreover, a recent study demonstrated that CsSEC23, a subunit of the COPII (coat protein complex II) vesicle, plays a regulatory role in controlling fruit glossiness in cucumber. The *cssec23* mutant exhibited a glossy peel phenotype, characterized by significantly reduced wax accumulation but increased cutin content compared to wild type (WT). In addition, the cuticle of *cssec23* mutant fruit was thinner than that of WT. Several genes involved in wax and cutin transport were upregulated in the *cssec23* mutant (Gao et al., 2023). Overall, the regulatory mechanism of fruit glossiness in cucumber involves a complex interplay of genetic pathways, environmental stimuli, and developmental signals that collectively influence cuticle development, including the biosynthesis and transport of wax and cutin.

### 5.2 The regulatory mechanism of fruit glossiness in tomato

As a model plant, tomato has been extensively studied, providing valuable insights into the intricate regulatory network controlling fruit glossiness (**Figure 4**). Key regulatory components include TFs, genes involved in wax/cutin biosynthesis, phytohormone signaling pathways, and microRNAs (Petit et al., 2016; Gupta et al., 2021; Liang et al., 2021; Bres et al., 2022). These elements coordinate cuticle formation and surface properties that ultimately shape the glossy or matt appearance of tomato fruits.

The tomato AP2/ERF family has been shown to regulate cuticle development in several studies. The first characterized member, WAX INDUCER1/SHINE1 (WIN1/SHN1), was shown to promote both cutin and wax biosynthesis, thereby enhancing leaf glossiness in Arabidopsis. Overexpressing *WIN1/SHN1* significantly upregulated the expression of genes involved in cutin and wax biosynthetic pathways (Kannangara et al., 2007). Subsequent research identified three Arabidopsis SHN orthologs in tomato, *SlSHN1*, *SlSHN2*, and *SlSHN3*. All three genes exhibited expression patterns associated with the fruit epidermis. *SlSHN1* displayed a more constitutive expression in the exocarp throughout fruit development, whereas *SlSHN2* and *SlSHN3* were highly expressed in the exocarp of immature green fruit (Shi et al., 2013). Overexpression of *SlSHN1* in tomato resulted in increased wax deposition on leaf epidermal tissues, leading to a shiny leaf phenotype. However, the impact of *SlSHN1* on fruit glossiness was not examined in this study (Al-Abdallat et al., 2014). Functional characterization revealed that overexpression of *SlSHN3* in Arabidopsis led to shiny, bright green rosette leaves. In contrast, silencing *SlSHN3* in tomato resulted in a glossy fruit phenotype, accompanied by downregulation of genes involved in cutin biosynthesis and epidermal patterning. This was associated with reduced accumulation of both cutin and wax components in the fruit cuticle (Shi et al., 2013). A recent study further confirmed the critical role of SlSHN2 in regulating fruit glossiness by identifying a point mutation responsible for the glossy phenotype of the *slshn2* mutant. This mutation resulted in a lysine-to-asparagine substitution within the highly conserved middle domain of SHN proteins. The *slshn2* mutant exhibited a significant reduction in cutin accumulation, while wax abundance and composition remained largely unchanged. In addition to altered cuticle thickness and mechanical properties, changes in epidermal cell patterning and cuticle polysaccharide composition were also observed. RNA-Seq analysis revealed that several genes involved in wax and cutin biosynthesis were downregulated in the *slshn2* mutant, highlighting the regulatory role of *SlSHN2* in cuticle formation and surface characteristics (Bres et al., 2022).

In addition to the AP2/ERF transcription factors, a member of the HD-ZIP IV transcription factor family, *CD2* (*CUTIN DEFICIENT2*), was identified as responsible for the glossy fruit surface phenotype observed in the tomato mutant *pe*. In the *pe* mutant, cutin monomer content was dramatically reduced, whereas the total wax load remained largely unchanged. However, significant alterations were observed in both wax and cutin composition (Nadakuduti et al., 2012).

Petit et al. (2014) investigated 16 glossy and 8 matt tomato mutants to elucidate the relationship between fruit glossiness and cuticle characteristics. They found that alterations in wax load and composition, cutin content and composition, as well as epidermal and cuticle architecture, were closely associated with fruit glossiness. Additionally, *SlGDSL2*, encoding a GDSL lipase, was mapped as the gene associated with cutin deficiency and the glossy fruit phenotype in the *P15C12* mutant line (Petit et al., 2014). In a follow-up study, one of the glossy mutants analyzed in this collection led to the identification of *SlGPAT6*, encoding glycerol-3-phosphate acyltransferase 6, as a key regulator of fruit glossiness in tomato. The loss-of-function mutant, *slgpat6-a*, exhibited enhanced surface glossiness and alterations in epidermal cell morphology. Cuticle thickness overlying the fruit epidermal cells was significantly reduced, and cutin accumulation was notably impaired. While the total wax load did not differ between *slgpat6-a* and WT fruits, distinct changes in wax composition were observed. Moreover, the expression levels of several genes involved in cutin biosynthesis and assembly, as well as wax biosynthesis, were significantly downregulated in *slgpat6-a* compared to WT (Petit et al., 2016).

Protein phosphatase 2Cs (PP2Cs) are important component of the ABA signaling pathway. In tomato, SlPP2C3 was recently shown to influence fruit glossiness by regulating cuticle composition in the outer epidermis through modulation of cutin and wax metabolism. Fruits from *SlPP2C3-RNAi* lines exhibited a matt fruit surface compared to WT. Scanning electron microscopy analysis revealed a warty outer epidermis in the *SlPP2C3-RNAi* lines, suggesting altered cuticle structure. RNA-Seq analysis further revealed the differential expression of numerous cuticle-related genes: several involved in wax and cutin biosynthesis, transport, and assembly were upregulated, while seven wax biosynthetic genes and one gene related to cutin biosynthesis were downregulated (Liang et al., 2021).

The microRNA-encoding gene *SlMIR164a* has been implicated in the development of the outer epidermis and cuticle in tomato (Gupta et al., 2021). CRISPR/Cas9-mediated knockout of *SlMIR164a* resulted in a less glossy appearance in *slmir164a* ^CR-21^ mutant fruits. Interestingly, these fruits exhibited significantly higher accumulation of both cutin and wax at the orange and red ripe stages, suggesting that the altered fruit surface phenotype was associated with irregularities in cuticle deposition and organization (Gupta et al., 2021). Taken together, these regulatory genes influence tomato fruit glossiness by modulating cuticle development, including the biosynthesis, transport, and assembly of wax and cutin. These processes collectively determine the quantity and composition of cuticular components, which ultimately shape the fruit’s surface appearance.

### 5.3 The regulatory mechanism of fruit glossiness in other species

In contrast to the extensive studies in cucumber and tomato, reports on genes regulating fruit glossiness in other species are relatively scarce, and their functions still require experimental validation. For example, a recent transcriptomic analysis by Wang et al. (2024) investigated eggplant peels with varying levels of glossiness and identified several candidate genes associated with this trait. Specifically, the long-chain acyl-CoA synthetase gene (*Smechr0102162*), ERF transcription factors (*Smechr0402179* and *Smechr0902337*), and C2H2 transcription factor (*Smechr0104003*) were found to be expressed at lower levels in glossy eggplant fruit peels compared to matt peels (Wang et al., 2024). Although several ERF and C2H2 transcription factors have been characterized as regulators of fruit glossiness in tomato and cucumber, the roles of their homologs in eggplant remain to be experimentally validated.

Previous studies on the ‘Newhall’ Navel orange (*Citrus sinensis* [L.] Osbeck) and its glossy mutant “glossy Newhall” revealed that numerous genes involved in wax biosynthesis and transport pathways were down-regulated in the glossy mutant. This down-regulation led to reduced wax accumulation and the loss of epicuticular wax crystals, ultimately resulting in the glossy appearance of “Glossy Newhall” fruits (Liu et al., 2012, 2015). Similarly, Wan et al. (2020) analyzed the glossy mutant “Gannan No. 1” and observed a significant reduction in wax content throughout fruit development (Wan et al., 2020). However, the specific genes responsible for the glossy phenotype in these citrus mutants remain unidentified.

β-ketoacyl-CoA synthase (KCS) is a key enzyme in VLCFA elongation. Recently, a total of 96 genes encoding KCS were identified across six *Citrinae* species. Among them, *CsKCS2* and *CsKCS11* were found to be highly expressed in the flavedo and showed a marked increase in expression during fruit ripening. Functional analysis in Arabidopsis demonstrated that overexpression of *CsKCS2* or *CsKCS11* significantly increased the accumulation of cuticular wax in leaves. These findings suggest that CsKCS2 and CsKCS11 may play important roles in fruit cuticular wax synthesis during ripening (Yang et al., 2021).

A similar study on bilberry (Vaccinium myrtillus L.) found that the glossy mutant displayed a significant decrease in the density of epicuticular wax crystals compared to WT fruit. The glossy phenotype was linked to alterations in the chemical composition of the wax, which in turn affected its morphology. Gene expression analysis indicated that wax biosynthesis and transport genes including CER26-like, CER3-like, LTP (lipid transfer protein), and BAS (β-Amyrin synthase), are involved in the regulation of fruit glossiness in bilberry (Trivedi et al., 2021). Interestingly, a grapevine (Vitis vinifera) TF has been identified as a regulator of fruit glossiness. Overexpression of the grapevine R2R3-MYB transcription factor VvMYB5b in tomato altered the shape and size of leaf epidermal cells and modified wax composition, collectively leading to enhanced fruit glossiness (Mahjoub et al., 2009). The functional analysis of VvMYB5b in grapevine remain to be conducted to further validated its regulatory role in fruit glossiness.

## 6 Conclusion and perspectives

Fruit glossiness represents a complex phenotype governed by multiple layers of regulation spanning cuticle composition, epidermal morphology, and environmental cues (**Figures 1**, **Figure 2**). As this review demonstrates, recent years have witnessed remarkable progress in elucidating the biochemical pathways and genetic networks responsible for gloss formation, particularly in cucumber and tomato (**Figure 4**). Several key regulatory genes, including biosynthetic enzymes, TFs, and components of the vesicle transport machinery, have been functionally characterized, providing a foundation for understanding gloss at the genetic and cellular levels (Shi et al., 2013; Liang et al., 2021; Gao et al., 2023; Hao et al., 2024).

**Figure 5:**
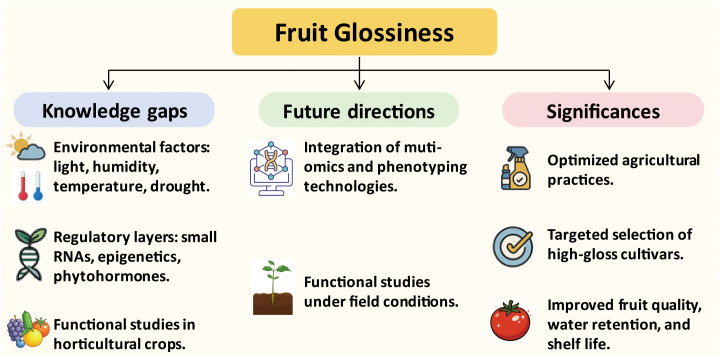
Summary of knowledge gaps, future directions, and benefits related to fruit glossiness. The left panel highlights major knowledge gaps, including the influence of environmental factors (e.g., light, humidity, temperature, and drought) and the limited understanding of regulatory layers such as small RNAs and epigenetic modifications. The middle panel outlines future research directions, emphasizing the integration of multi-omics technologies and the need for functional studies under field-relevant conditions. The right panel summarizes the potential benefits of advancing gloss-related research, including the optimization of agricultural practices, targeted selection of high-gloss cultivars, and improvements in fruit quality, water retention, and shelf life.

Nevertheless, significant knowledge gaps remain. The precise mechanisms by which cutin and wax components are spatially deposited and organized to modulate surface optical properties are not fully understood (**Figure 5**). In particular, the mechanisms underlying gloss variability in response to environmental factors, such as humidity, light, drought, and temperature, remain largely unexplored. This knowledge is essential for understanding how fruit surface properties are modulated in real-world agricultural settings. Moreover, the regulatory roles of microRNAs, long non-coding RNAs, epigenetic factors such as DNA methylation and histone acetylation, as well as phytohormone signaling in fine-tuning of gene expression during cuticle development and gloss formation are still poorly understood. These regulatory layers could represent crucial control points that integrate developmental cues and environmental stimuli. Another major challenge lies in extending findings from model systems to a broader array of horticultural crops, many of which exhibit unique cuticle features and gloss characteristics. Functional studies in these species remain limited due to technical constraints in transformation and gene editing.

To address these challenges, future research should prioritize the integration of multi-omics approaches, such as transcriptomics, metabolomics, and lipidomics, with high-throughput, quantitative phenotyping technologies to decipher the dynamic regulation of fruit glossiness across developmental stages and environmental conditions. Additionally, functional studies under controlled and field-relevant environments are essential to decipher how abiotic factors influence cuticle development and glossiness (**Figure 5**).

Importantly, advancing our understanding of the molecular mechanisms behind fruit glossiness holds significant potential for practical applications in agriculture and breeding (**Figure 5**). The glossy phenotype is not only visually appealing to consumers but also associated with fruit quality, water loss regulation, and postharvest shelf life. Optimized agricultural practices, such as hormone treatments and environmental controls, can help enhance fruit glossiness, offering practical benefits to growers. By identifying key regulatory genes and developing molecular markers for gloss-related traits, breeders can accelerate the selection of cultivars with improved surface aesthetics and physiological performance. Genome editing technologies can be employed to fine-tune gloss-related pathways with high precision, allowing for targeted trait improvement. In conclusion, a more refined and systems-level understanding of fruit glossiness will not only enrich our fundamental knowledge of cuticle biology but also bridge the gap between basic research and practical breeding. Such insights will support the development of high-gloss cultivars with higher market values.
